# Moderate-intensity aerobic and resistance exercise is safe and favorably influences body composition in patients with quiescent Inflammatory Bowel Disease: a randomized controlled cross-over trial

**DOI:** 10.1186/s12876-019-0952-x

**Published:** 2019-02-12

**Authors:** Owen Cronin, Wiley Barton, Carthage Moran, Donal Sheehan, Ronan Whiston, Helena Nugent, Yvonne McCarthy, Catherine B. Molloy, Orla O’Sullivan, Paul D. Cotter, Michael G. Molloy, Fergus Shanahan

**Affiliations:** 1 Department of Medicine, National University of Ireland, University College Cork, Cork University Hospital, Wilton, T12 DC4A, Cork, Ireland; 2 APC Microbiome Ireland, National University of Ireland, Cork, T12 YT20 Ireland; 3 Teagasc Food Research Centre, Moorepark, Fermoy, P61 C996 Cork, Ireland

**Keywords:** Clinical trials, Microbiome, Exercise, Body composition

## Abstract

**Background:**

Overweight and metabolic problems now add to the burden of illness in patients with Inflammatory Bowel Disease. We aimed to determine if a program of aerobic and resistance exercise could safely achieve body composition changes in patients with Inflammatory Bowel Disease.

**Methods:**

A randomized, cross-over trial of eight weeks combined aerobic and resistance training on body composition assessed by Dual Energy X-ray Absorptiometry was performed. Patients in clinical remission and physically inactive with a mean age of 25 ± 6.5 years and Body Mass Index of 28.9 ± 3.8 were recruited from a dedicated Inflammatory Bowel Disease clinic. Serum cytokines were quantified, and microbiota assessed using metagenomic sequencing.

**Results:**

Improved physical fitness was demonstrated in the exercise group by increases in median estimated VO_2max_ (Baseline: 43.41mls/kg/min; post-intervention: 46.01mls/kg/min; *p* = 0.03). Improvement in body composition was achieved by the intervention group (*n* = 13) with a median decrease of 2.1% body fat compared with a non-exercising group (*n* = 7) (0.1% increase; *p* = 0.022). Lean tissue mass increased by a median of 1.59 kg and fat mass decreased by a median of 1.52 kg in the exercising group. No patients experienced a deterioration in disease activity scores during the exercise intervention. No clinically significant alterations in the α- and β-diversity of gut microbiota and associated metabolic pathways were evident.

**Conclusions:**

Moderate-intensity combined aerobic and resistance training is safe in physically unfit patients with quiescent Inflammatory Bowel Disease and can quickly achieve favourable body compositional changes without adverse effects.

**Trial registration:**

The study was registered at ClinicalTrials.gov; Trial number: NCT02463916.

**Electronic supplementary material:**

The online version of this article (10.1186/s12876-019-0952-x) contains supplementary material, which is available to authorized users.

## Background

Contrary to the classical phenotype of low body weight and malnourishment from earlier times, many patients with Inflammatory Bowel Disease (IBD) are now overweight or obese [1]. This increases the risk of metabolic disorders such as type two diabetes mellitus and non-alcoholic fatty liver disease [2]. While some patients with IBD experience cachexia, particularly those with advanced Crohn’s disease, modern lifestyles typified by sedentary living and high-caloric food availability, are contributing to the increase in obesity and obesity-related conditions in patients with IBD.

Optimal control of inflammatory activity remains a primary objective in the treatment of IBD and is an area in which considerable advances have taken place in the past decade. However, attention to details such as the consequences of physical activity through exercise has received less attention. Known for its anti-inflammatory and multi-organ metabolic effects [3, 4], exercise represents first line treatment for patients with type two diabetes mellitus and non-alcoholic fatty liver disease [5, 6]. Potential benefits of exercise for patients with IBD extend beyond metabolic enhancement and include improvements in quality of life, fatigue levels and bone mineral density. However, improvement in body composition through muscle mass accretion remains an underexplored area that could exert a particularly advantageous effect of exercise in patients with IBD.

Habitual levels of physical activity in patients with IBD are significantly lower than in matched controls [7]. When combined with nutrient malabsorption and corticosteroid treatment, this leads to a higher prevalence of sarcopenia in IBD sufferers. Despite improvements in pharmacological treatments, sarcopenia prevalence in IBD populations remains high (up to 60% in Crohn’s Disease) [8], irrespective of BMI [9], and predicts future need for surgical treatment in both underweight [10] and overweight/obese cohorts [9].

Notwithstanding the likely benefits of exercise in IBD, there is a paucity of focussed prospective studies [11]. No trials have examined the efficacy or safety of combined aerobic and resistance (strength) training in improving body composition and increasing muscle mass in patients with IBD. Therefore we addressed this issue in a randomized, controlled cross-over trial to determine if a combined aerobic and resistance exercise program could safely and quickly achieve desirable body compositional changes in patients with IBD.

## Methods

### Study design and conduct

**Fig. 1:**
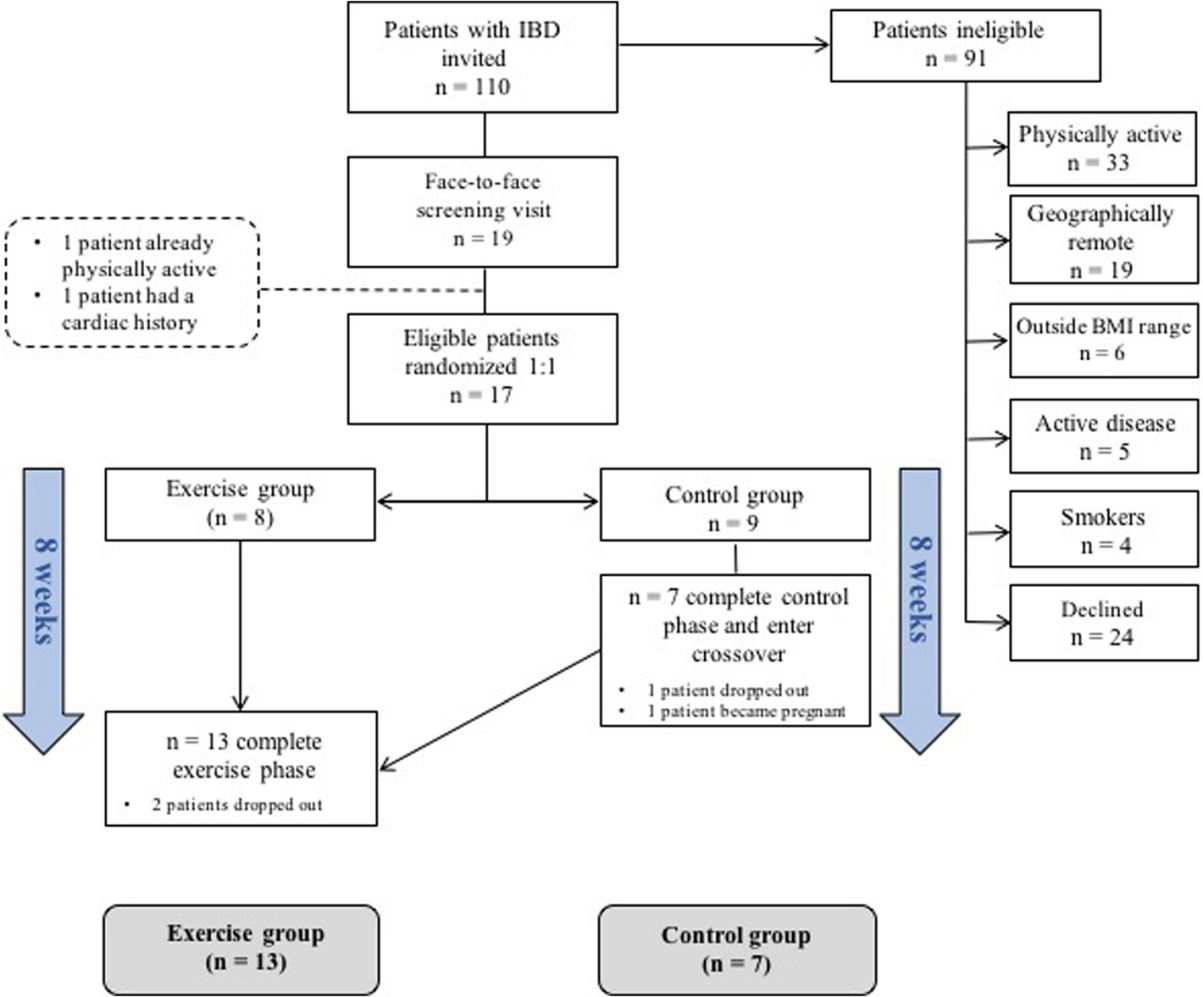
Study outline indicating the number of participants at each stage of the study and reasons for exclusion from the trial

We performed a single-centre, randomized, partial cross-over trial recruiting between March and December 2015 (Fig. 1). The trial was registered at https://clinicalTrials.gov (Trial number: NCT02463916) and conforms to CONSORT guidelines for randomized controlled trials. Prior to trial commencement, ethical approval was granted by the Clinical Research Ethics Committee of the Cork Teaching Hospitals. All volunteers gave written informed consent. The clinical notes of age-suitable patients attending a dedicated Inflammatory Bowel Disease outpatient clinic at Cork University Hospital, Cork City, Ireland, were reviewed by the study investigators for exclusion criteria. If no reason for exclusion was apparent, consecutive patients were invited to participate at the clinic, by letter or by telephone. Patients were invited to participate according to a strict, eligibility-based criteria as outlined below.

Eligible patients were randomized (1:1) by simple methods (drawing of group numbers from a concealed container) to one of two groups: Exercise or Control. Randomization was overseen by two study investigators. Due to the nature of the intervention, a combined exercise program, it was not possible to conceal group allocation to the participants or investigators. Patients randomized to the exercise group underwent measurement and sampling eight weeks apart (start and end of the intervention period). During this intervention period, participants in the exercise group undertook a combined aerobic and resistance exercise program. Patients randomized to the non-exercising control group initially underwent a controlled-phase where usual levels of physical inactivity were maintained for eight weeks. Participants were measured for primary and secondary outcomes at the start and end of this period. Following completion of the control phase, participants then ‘crossed over’ to the intervention arm of the study, where they undertook the same exercise program for eight weeks (Fig. 1). Volunteers were again measured at the end of the eight-week intervention period.

### Participants

Male and female patients, aged 18 to 40 years were recruited from a dedicated IBD clinic. All patients, including those assigned to the control arm of the study, were required to have a diagnosis of IBD. Eligible patients were required to be physically inactive or have low levels of activity (as defined by the International Physical Activity Questionnaire) [12] with no involvement in regular or organised exercise in the month prior to recruitment. Furthermore, only patients in disease remission and with a BMI of 22 to 35 kg/m^2^ were eligible. Any patients having experienced a disease flare or having received treatment with corticosteroids in the two weeks prior to invitation were excluded. Volunteers who had received oral antibiotics, bowel preparation, or had suffered an infectious gastrointestinal illness in the last month were also excluded. Patients with a history of total colectomy were excluded.

Provided eligibility was met, patients were then required to attend a face-to face screening visit at the study site (Department of Medicine, Cork University Hospital, Ireland). Here, patients underwent further screening and a medical evaluation to ensure safe participation in the exercise program using an adapted version of the American College of Sports Medicine’s safe participation questionnaire [13]. Past or current medical history of co-morbidities such as cardiovascular disease or diabetes mellitus excluded patients from participation.

### Intervention and control

Patients randomized to the control phase of the study were instructed to maintain usual levels of physical activity (none or low) for the eight-week period. Participants assigned to the exercise arm of the study, primarily or following cross-over, participated in the same 8-week combined aerobic and resistance training program. This exercise was moderate in intensity and progressive, based on the couch to 5 km training programs. Participants received free gymnasium membership at the Mardyke Arena at University College Cork, Ireland, for the duration of the exercise intervention period and were required to attend three times per week for eight weeks. Before commencement, participants were familiarized with the training program during a 90-min induction session with a study-specific gym instructor. This included demonstration and practice with all aerobic and resistance training equipment. Practical support was provided for participants throughout by the designated gym staff and study investigators. Each participant was measured for individual differences in range of motion on the resistance training machines. The gym instructor observed the patients using the machines and provided correction and feedback where necessary. For resistance machines, one repetition maximum (1RM) values were calculated using the Brzycki formula [14].

The outline of the exercise program used is identical to that described by the authors previously [15]. Volunteers were reminded of the desired intensities on each of their weekly exercise training program print-outs. Likewise, compliance with the prescribed exercise program was reviewed remotely by investigators using the FitLinxx® activity monitoring system (Shelton, Connecticut, USA) as outlined previously [15]. Prior to study commencement, the gym facility’s FitLinxx® software and hardware was re-calibrated and regularly maintained throughout.

### Measurements and efficacy criteria

The primary outcome was change in participant body composition as determined by Dual Energy X-ray Absorptiometry (DEXA). A GE Healthcare Lunar iDXA machine (Madison, Wisconsin, USA) at the Bone Densitometry Unit, Cork University Hospital was used. The enCORE software (V.13.4, 2010) analysed body composition using a three-compartment model (fat mass, bone mass, lean tissue). Total fat mass, truncal fat mass, total lean tissue mass and percentage body fat were recorded. Scanning was performed with the volunteers dressed in light clothing with removal of metal-wear and after they had voided urine. To reduce potential interference with true body composition assessment and the effects of diurnal variation, patients were measured between 7 a.m. and 10.30 a.m. and were asked to refrain from alcohol and moderate to vigorous physical activity for at least 24 h prior to measurement. Quality control analysis was performed daily on the iDXA machine.

Secondary outcomes included changes in disease activity scores, quality of life measures, anxiety and depression indices, serum pro-inflammatory cytokine levels, and change in the α- and β- diversity of the gut microbiome. For patients with Crohn’s Disease the Harvey Bradshaw Index was used to assess disease at the start and end of the exercise and control periods [16]. For patients with Ulcerative Colitis, the Simple Colitis Index was used [17]. Patients were instructed to report any deterioration in gastrointestinal symptoms to the study team and any symptoms suggestive of a disease flare meant removal of the patient from the study. Quality of life was assessed using the Short Form 36 health survey (SF36®V2) [18] at the start and end of the study periods. Similarly, the Hospital Anxiety and Depression Scale (HADS) [19], State-Trait Anxiety Inventory (STAI) [20], and the Beck Depression Inventory II (BDI-II) [21] were used to assess changes in mood score and psychological well-being.

To assess improvements in physical fitness following the intervention, all exercising patients underwent cardiorespiratory fitness testing before and after the exercise program. To avoid harm from unaccustomed vigorous exercise, we used a validated, submaximal assessment of peak aerobic capacity to estimate maximal oxygen uptake (VO_2max_) [22]. The Rockport one-mile walk test was performed at the indoor running track of the Mardyke Arena, Cork. This testing took place in a standardized temperature environment.

### Pro-inflammatory cytokine measurement

All study participants underwent phlebotomy in the resting state before and after the exercise and control periods. Approximately 2mls of supernatant sera were harvested by pipette, frozen and stored at − 80 °C in polypropylene cryogenic vials. Following collection of all serum samples, resting pro-inflammatory cytokines were measured using an electrochemiluminesence-based solid-phase multiplex assay (MSD; Meso Scale Discovery platform, Rockville, Maryland, USA). Serum concentrations of interleukins 6, 8, and 10; and Tumour Necrosis Factor-α (TNF-α) were measured. The lower limit of detection for the assays was less than 1 pg/ml and standardized calibration curves were constructed for each plate. Samples were measured in duplicate and the mean cytokine concentration (pg/ml) of the duplicates was used for the subsequent statistical analysis.

### DNA extraction and metagenomic sequencing of faecal microbiome

Patients’ stool samples were collected into a sealed, secure container and transported to the Teagasc Moorepark research facility for DNA extraction. DNA was extracted from fresh faecal material before freezing and within 6 h of defecation in the vast majority of cases and never after 12 h. Extraction was performed using a QIAmp DNA stool minikit (Qiagen, Crawley, West Sussex, United Kingdom). Faecal sample preparation prior to DNA extraction and DNA library preparation was identical to methods previously described by the authors [15]. An equimolar library pool of all samples was made prior to sequencing on an Illumina NextSeq 500 (chemistry V.2.0) sequencing platform (Teagasc sequencing facility). High-throughput sequencing was performed using the high-output 500/550 reagent kit.

### Bioinformatic processing of microbial metagenomic sequencing

Quality control of metagenomic FASTQ sequences proceeded with the removal of host (human) reads using NCBI Best Match Tagger (BMTagger version 1.1.0). Reads were converted to Binary Alignment Map (BAM) format and sorted using FastqToSam (version 2.7.1). Low-quality reads (Phred quality score < 20), adapter sequences and short reads (Length cutoff: 105 bp) were trimmed using trimBWAstyle.usingBam.pl script. PCR duplicates were removed using MarkDuplicates from Picard tools version 2.7.1. Finally, forward and reverse reads were merged and converted to FASTA format using IDBA fq2fa version 1.1.1. The Human Microbiome Project (HMP) Unified Metabolic Analysis Network (HUMAnN2 V.0.99) pipeline was used to conduct a functional profile of high-quality reads [23]. Microbial metabolic pathway models produced by HUMAnN2 were derived from the MetaCyc database [24] and formed the analyses performed on microbial metabolic profiling. Taxonomic profiling was facilitated with the Kaiju taxonomic assignment software tool (V.1.5.0).

### Sample size and statistical analysis

To detect a 2% reduction in body fat percentage after an eight-week intervention, with a two-sided significance level of 5 and 80% power, we calculated a requirement of seven patients in the control group and 14 in the exercise group (presuming no loss to follow-up). For statistical analysis and comparison, patients and their associated data were grouped according to exercise and control groups. The relevant data of participants completing the cross-over phase of the trial were compiled with the exercise group. The Statistical Package for the Social Sciences V.23 (SPSS Inc., Chicago, Illinois, USA) and the R statistical programming environment (V.3.3.2) were used for the statistical analysis. As the majority of data was non-normally distributed, non-parametric analyses were conducted to compare the groups at baseline. Medians and interquartile ranges (IQR) are used unless otherwise stated. Similarly, non-parametric statistical tests were employed in the analysis of microbiome data. Primary and secondary analyses were conducted on a per protocol base. A type I error rate ≤ 0.05 was considered significant in all cases. Adjustment of significant *p*-values for multiple testing was performed using the Bonferroni test [25].

The adonis2 function in the vegan R package (V.2.4–3) was used to statistically assess dissimilarity matrices (Bray-Curtis) derived from microbial data [26]. Identification of statistically relevant taxonomic features were identified using the analysis of composition of microbiomes (ANCOM) test, implemented in the R package (V.1.1–3) [27]. Measurements of α-diversity and calculations of relative abundances were also performed with the vegan package. Relative-abundance data were generated separately for identified species within each phylogenetic domain (e.g. Bacteria). Correction of p-values relating to microbiome analysis was performed using the Benjamini-Hochberg false-discovery rate (FDR) [28] in the base *stats* package in R.

### Data and software availability

The microbial DNA sequences have been deposited in the European Nucleotide Database (ENA) database under ID code PRJEB27623.

## Results

### Study participants

One hundred and ten patients aged 18 to 40 years were invited to participate in the trial by letter, phone or in person at the IBD clinic. Of these, 19 patients were eligible for enrolment (exclusion reasons specified in Fig. 1). These patients attended a face-to-face screening visit and two further patients were excluded based on their high level of physical activity and their family history of early coronary artery disease, respectively. Therefore, 17 patients, with a mean age of 25 ± 6.5 years and BMI of 28.9 kg/m^2^ ± 3.8, were randomly allocated to the study arms. Patient characteristics are described in Table 1 and indicate no significant differences in clinical variables at baseline between the two groups, including body composition parameters. Of the 9 patients assigned to the control phase of the study, seven patients completed the eight-week control period and subsequently crossed over to the exercise arm of the study. Of the 15 participants entering the exercise arm of the study (8 directly randomized and seven cross-overs), 13 completed the exercise program. For the two patients who did not complete the exercise program, one dropped out due to personal reasons and the other due to time constraints.

**Table 1:** Baseline demographic, clinical and anthropometric characteristics of patients in the exercise and non-exercising disease control groups

	Exercise group (*n* = 13)	Control group (*n* = 7)	*p*-value
Age (years)	33 (31, 36)	31 (31, 36)	0.938
Sex (female)	*n* = 4 (30.8%)	n = 1 (14.3%)	0.417^a^
Height (cm)	172 (165, 179)	173 (167, 183)	0.485
Weight (kg)	84.5 (75.8, 97)	89 (70.5, 102.9)	0.757
BMI (kg/m^2^)	28.1 (26.2, 32.4)	27.2 (24.5, 33.7)	0.938
Resting heart rate (Beats per minute)	73 (64, 77)	76 (58, 83)	0.817
Systolic Blood Pressure (mmHg)	123 (114, 131)	124 (119, 130)	0.485
Diastolic Blood Pressure (mmHg)	73 (69, 86)	82 (70, 86)	0.311
Body fat (%)	35.2 (30.9, 37.4)	34.2 (32.2, 35.7)	0.817
Fat mass - total (kg)	28.1 (22.8, 35.7)	29.9 (23.8, 34.7)	0.938
Fat mass - trunk (kg)	14.7 (12.9, 21.3)	16.8 (13.1, 22.2)	0.699
Lean tissue mass (kg)	50.2 (44, 61.6)	52.69 (45.8, 63.8)	0.643
Estimated weekly physical activity (kCals)	1017 (283, 1399)	266.9 (0, 1039)	0.097

Median values (inter-quartile ranges) are stated. Mann-Whitney U or Chi-squared test used to compare groups as appropriate. ^a^Indicates Chi-squared test

Enrolled patients were predominantly overweight or obese with a median baseline percentage body fat of 35%. Disease activity assessment scores were low at study entry (Table 2). However, approximately half of the trial participants had required a treatment course of corticosteroids in the year leading up to the study. All patients were receiving some form of disease maintenance therapy including 15% on anti-TNF-α therapy.

**Table 2:** Disease characteristics, baseline disease activity scores and pharmacological treatment of patients in the exercise and non-exercising disease control groups

	Exercise group (*n* = 13)	Control group (*n* = 7)	*p*-value
Condition (%)			
Ulcerative Colitis	8 (61.5%)	5 (71.4%)	0.658^a^
Crohn’s Disease	5 (38.5%)	2 (28.6%)	
Age at diagnosis (years)	25 (25, 31)	26 (22, 31)	0.642
Previous surgery for condition	Yes: 1 (7.7%)	Yes: 0	0.452^a^
Baseline Disease activity score			
Simple Colitis Index	1.5 (1, 2.75)	1 (0, 4)	0.833
Harvey Bradshaw Index	1 (0, 1.5)	1 (0, 1)	1.00
Disease treatment			
Current steroid use	None	None	
Steroid use in the last year (%)	Yes: 5 (38.5%) No: 8 (61.5%)	Yes: 3 (42.9%) No: 4 (57.1%)	0.848^a^
Current anti-TNF treatment	Yes: 2 (15.4%) No: 11 (84.6%)	Yes: 1 (14.3%) No: 6 (85.7%)	0.948^a^
Currently on immunomodulation	Yes: 100%	Yes: 100%	
Azathioprine	N = 1	N = 1	
Mesalazine	*N* = 11	*N* = 6	
6-mercaptopurine	N = 4	*N* = 2	
Proton pump inhibitors	N = 4	N = 4	

Proportions, medians (interquartile ranges) are stated as appropriate. Mann-Whitney U and Chi-squared tests performed to compare groups. ^a^Indicates Chi-squared test

Compliance with the prescribed exercise program was high with exercise group participants attending 87.5% of gym sessions (averaging 21 out of 24 sessions over 8 weeks). FitLinxx® data recorded that patients in the exercise group spent a mean 689 ± 113 min in aerobic exercise over the eight weeks, with an estimated 6813 ± 1952 cal expended during this period. Participants performed a mean of 4782 ± 812 weight repetitions during the intervention. Following trial completion, patients in the exercise group experienced a significant improvement in cardiorespiratory fitness, as measured by estimated VO_2max_ (Pre-intervention: 43.41mls/kg/min; post-intervention: 46.01mls/kg/min; *p* = 0.03, Wilcoxon signed-rank test).

### Primary outcome

**Fig. 2:**
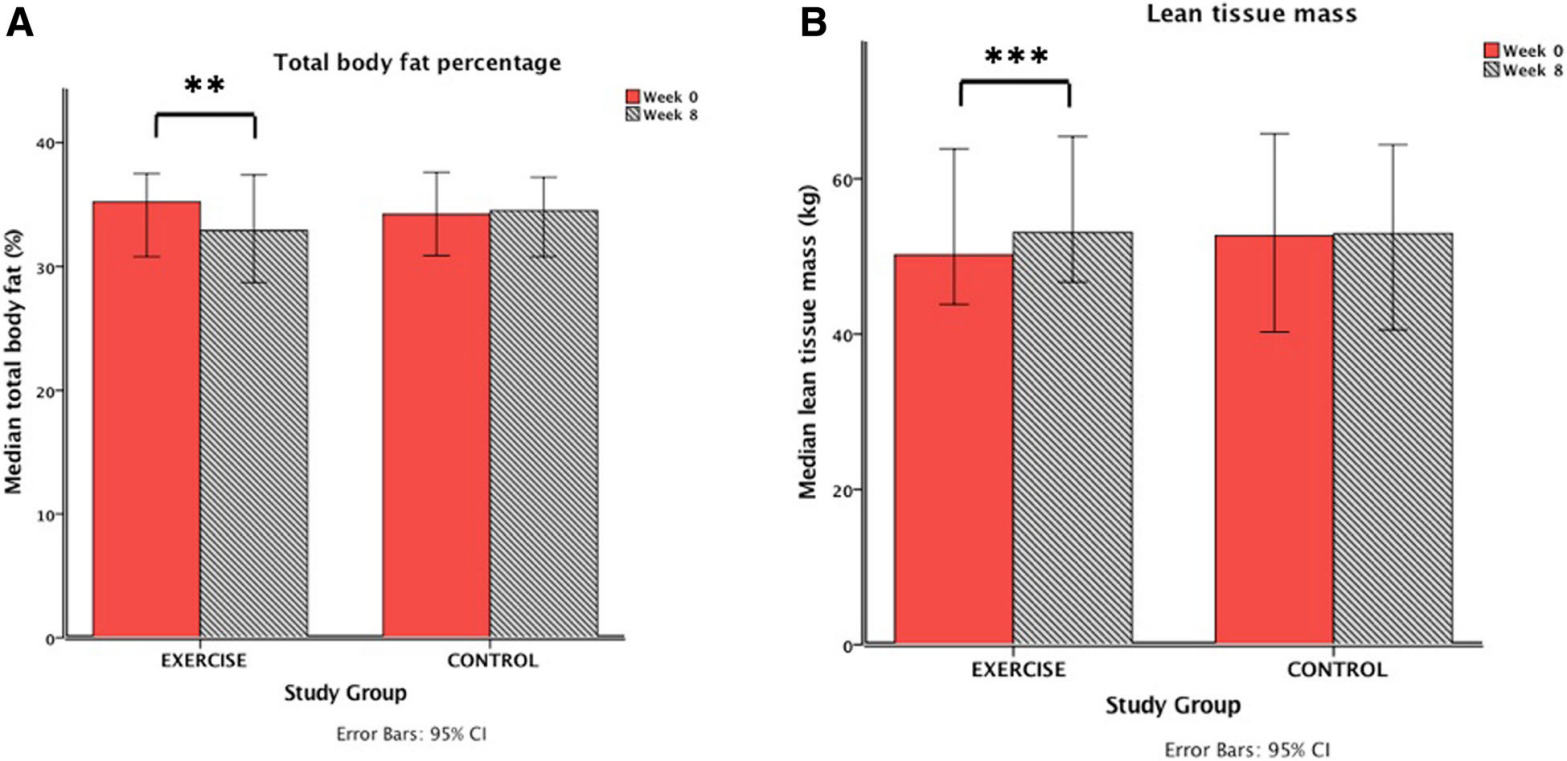
Comparison of body composition parameters (**a**: Total body fat percentage; **b**: Total lean tissue mass) before and after the combined aerobic and resistance exercise intervention in the EXERCISE group and in the non-exercising disease CONTROL group. ** Denotes *p*-value < 0.01 and ***denotes p-value < 0.001 as per Wilcoxon signed-rank test. Error bars denote 95% confidence intervals

The primary efficacy criterion (body composition change) was achieved in the exercise group after eight-weeks of combined aerobic and resistance training (Fig. 2, Table 3). Participants in the exercise group experienced favourable changes, with a median (IQR) 2.1% (− 2.15, − 0.45) decrease in total percentage body fat compared to a median gain of 0.1% (− 0.4, 1) body fat in the control participants (*p* = 0.022). Likewise, patients in the exercise group experienced a median 1.59 kg (0.68, 2.69) increase in total lean tissue mass compared to a decrease of 1.38 kg (− 2.45, 0.26) in the control group (*p* = 0.003).

**Table 3:** Change in clinical variables (Δ), body composition parameters and disease activity scores following exercise and control periods (8 weeks)

	Exercise group (*n* = 13)	Control group (*n* = 7)	*p*-value
Weight (kg)	0 (−1.15, 1.55)	−1.6 (−2.1, 0.2)	0.624
BMI (Kg/m^2^)	0 (−0.395, 0.48)	−0.58 (−0.74, −0.54)	0.116
Systolic blood pressure (mmHg)	−5 (− 12, − 2)	−8 (− 14, − 2)	0.536
Diastolic blood pressure (mmHg)	−4 (−8.5, 0.5)	−9 (− 10, − 2)	0.311
Resting heart rate (Beats per minute)	−7 (− 12, − 2)	−1 (−9, 6)	0.211
Total body fat percentage (%)	−2.1 (− 2.15, − 0.45)	0.1 (− 0.4, 1)	0.022^a^
Fat mass - total (kg)	− 1.52 (− 2.32, − 0.04)	− 0.04 (− 0.93, − 0.04)	0.487
Fat mass - trunk (kg)	−0.82 (− 1.4, − 0.002)	−0.13 (− 0.8, 0.78)	0.688
Lean tissue mass(kg)	1.59 (0.68, 2.69)	−1.38 (− 2.45, 0.26)	0.003^a^
Change in disease activity score			
Simple Colitis Index	−1 (− 1, 0)	0 (− 1.5–2)	0.435
Harvey Bradshaw Index	0 (− 1, 0.5)	0 (0, 0)	1.00

Mann-Whitney U tests were used to compare changes in variables between groups. Median values for the change in these variables (interquartile ranges) are stated. Significant p-values adjusted for multiple comparisons (Bonferroni). ^a^indicates statistical significance

### Secondary outcomes

#### Disease activity, quality of life and mood scores

There was no significant deterioration or improvement in disease activity scores in the intervention group (Table 3). Participants’ disease activity indices remained low after 8 weeks of exercise and no patient was removed from the trial due to a flare of symptoms. Two patients, one from each group, required oral antibiotics during the study for mild, intercurrent, non-IBD related infections (1 superficial skin infection and 1 lower respiratory tract infection). These incidents occurred at the midpoint of the study (weeks four and five).

There were no statistically significant changes in quality of life scores (SF36®V2, four physical and four mental health domains) or mood and anxiety scores (HADS, STAI, BDI-II) between the control and intervention groups after eight-weeks (data not shown).

#### Pro-inflammatory cytokines

Pro-inflammatory cytokines (IL-8, IL-10, IL-6 and TNF-α) and circulating levels of C-reactive protein (CRP) were similar at baseline in both groups indicating low levels of disease activity and there was no deterioration after the intervention suggesting disease stability with exercise (Additional file 1: Table S1).

#### Metagenomic assessment of gut microbiota

**Fig. 3:**
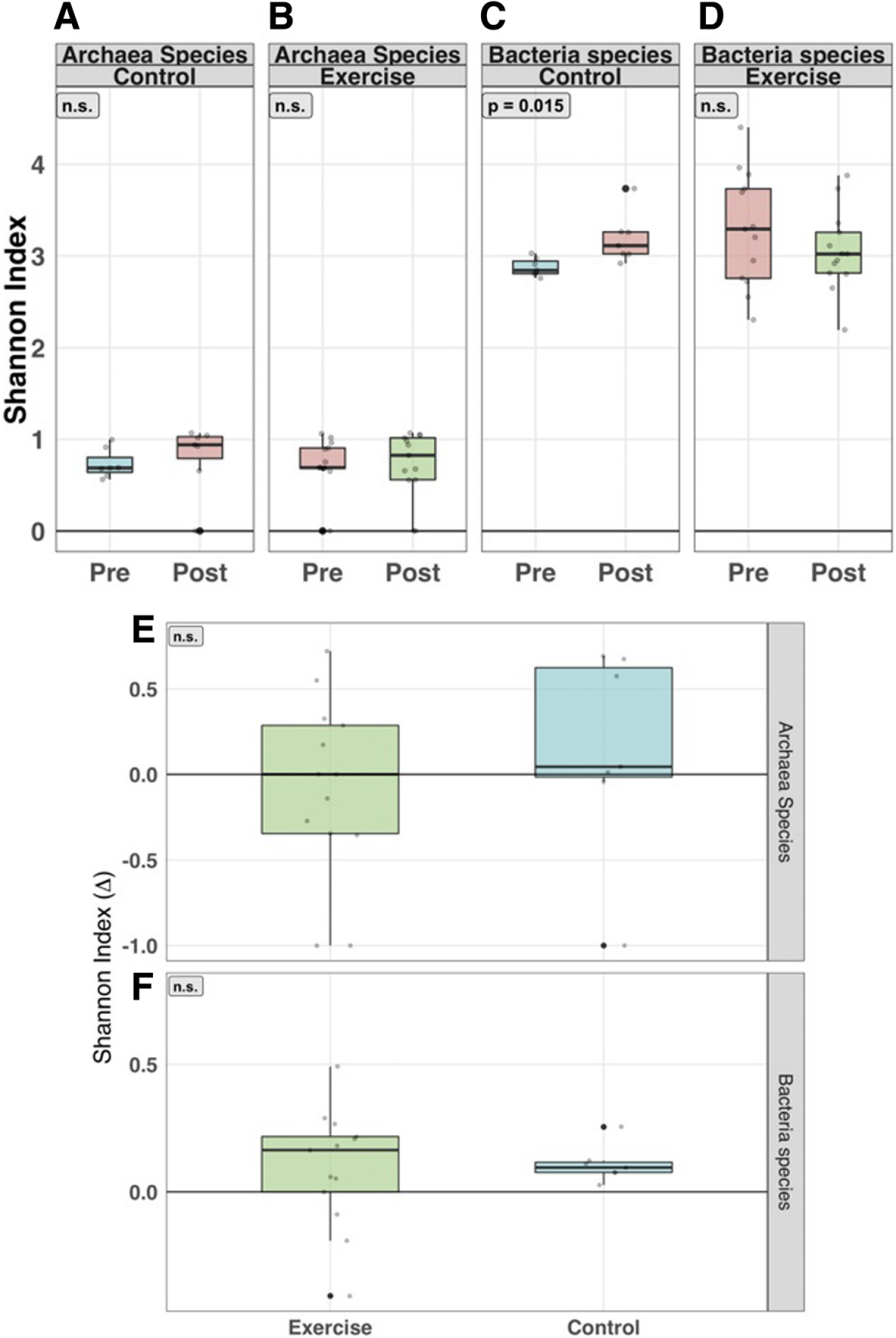
α-diversity of taxonomic profiling. (**a**-**d**) Shannon α-diversity H-index of Bacteria and Archaea species at week 0 (pre) and week 8 (post). (**a** and **b**) Subtle median increases of Archaea α-diversity for patients in both treatment. (**c** and **d**) Bacterial α-diversity was significantly raised in the control group (*p*-value = 0.015), while a moderate median decrease was shown in the exercise group. (**e**) Percent change (Δ) of α-diversity for Archaea species shows a slight increase in the control group compared to the exercise group (non-significant). (**f**) Both exercise and control groups show an increase in α-diversity for Bacteria species. *P*-values were calculated from the Wilcoxon signed-rank test

There was a modest, but not statistically significant increase in α-diversity (intra-individual) of *Archaea* species after the study period (Fig. 3a and b). Mean α-diversity of bacterial species increased in participants following the control period (*p* = 0.015, Fig. 3c), however comparisons of within-group alterations of α-diversity (percent Δ) showed no significant differences between exercise and control groups (Fig. 3e and f). Similarly, no major shifts in taxonomic β-diversity (inter-individual) were detected for bacterial species (Additional file 2: Figure S1A & B), archaeal species (Additional file 2: Figure S1C & D), or viral species across both groups (data not presented).

Metabolic pathways generated from metagenomic data were compared and illustrated subtle, non-significant differences within the groups (i.e., week 0 vs. week 8) and across groups. No major, statistically significant changes were elucidated when α-diversity (Shannon index) and taxonomic β-diversity of generic and species-specific pathway models were assessed (Additional file 3: Figure S2A to Additional file 4: Figure S3D inclusive).

## Discussion

### Main findings and relevance

The results show that favourable alterations in body composition can be achieved over a relatively short period of time with combined aerobic and resistance exercise in patients with IBD. This was feasible in sedentary patients and without any deterioration in disease activity or levels of pro-inflammatory cytokines. It is possible that exercise had additional anti-inflammatory properties that were not assessed in this study. This is highly likely given the emerging evidence for exercise-induced suppression of inflammation [4, 29]. The importance of the finding lies in the metabolic benefits of improving long-term body composition given the increasing risk of obesity and metabolic complications in patients with IBD [30, 31]. This study should reassure patients and clinicians that exercise is safe and desirable and can quickly lead to objectively verifiable improvements in lean tissue mass. Reversing or preventing sarcopenia in patients with IBD, irrespective of BMI, is an important goal [9, 10].

This prospective trial also provided us with the opportunity to explore the influence of exercise on the gut microbiome of patients with IBD. Recently, research is converging on the ability of physical activity omultiple facets of the hepatobiliary-gut axis including the gut microbiota and bile acid metabolism [32]. Reassuringly, and contrary to some evidence that exercise has the potential to aggravate gut symptomatology [33], combined aerobic and resistance exercise of moderate-intensity did not have detrimental effects on the composition or diversity of the gut microbiome. The results demonstrate similar findings to our previous study in healthy but physically inactive volunteers, where only modest changes in the composition and functional capacity of the gut microbiome occurred following a short-term period of fitness improvement through an identical moderate exercise program [15]. It is important to distinguish the effects of short term exercise from the physiological changes that occur with sustained fitness or prolonged high-intensity exercise. This remains to be assessed in patients with IBD [6]. Indeed, high-intensity exercise may lead to superior metabolic benefits and might induce favourable changes in the bacterial microbiome in patients with IBD, similar to what has been observed in professional athletes [34, 35].

### Comparison with other studies

The majority of exercise-intervention studies in patients with IBD have focused on low-impact, aerobic activities such as walking and almost all studies have been uncontrolled [7]. One randomized controlled trial of moderate intensity outdoor running (three times per week for 10 weeks) demonstrated improved quality of life measures in patients with mild to moderately active Crohn’s and Colitis. No adverse events or worsening of symptoms were reported over a 10-week period [36], supporting efforts to recommend physical activity for patients with IBD [11].

Despite established evidence for resistance training in cancer-related sarcopenia [37], only one, non-randomized study has examined strength training in IBD reporting good compliance and improvement in muscle strength with the prescribed exercise program (thrice-weekly, 12 resistance exercises at 60–70% of one repetition maximum) [38]. There was good compliance with the exercise programme in the present study which is the first randomized controlled trial to examine the role of resistance training in patients with IBD.

### Strengths and limitations

The strengths of this study are several-fold. The cohort of patients examined in this trial represents a group who can harness meaningful benefit from lifestyle interventions to improve their metabolic health. At recruitment, patients in this study were unfit, predominantly overweight or obese, with high body fat percentages, placing them at risk of future metabolic disorders. Following intervention, this cohort successfully demonstrated improvements in body composition in a short period of time. Despite their disease inactivity at study entry, a large proportion of study participants (almost half) had required corticosteroids for disease flare in the previous 12 months, and all patients were on disease-controlling medication. This indicates that patients at risk of disease flare in the near-future can also benefit from body composition improvement with exercise and engage in combined exercise programs without undue concern for their symptoms. The cross-over design of this study also represents a strength of this study, reducing the impact that additional contributory variables (e.g. age, gender, previous exercise history) may have on the primary outcome response to exercise intervention by homogenizing these factors in the exercising and non-exercising control groups. This is a recognized advantage of this type of trial design [39].

The study was limited to patients in remission. Other limitations relate to the relatively short-term follow up and sample size of the group which may have been underpowered to detect meaningful changes in secondary outcomes. Future studies should be extended to those patients with co-existing IBD and metabolic disorders including non-alcoholic fatty liver disease and type two diabetes, who may benefit the most from exercise. Furthermore, although analysis of the gut microbiome was not the primary endpoint of interest, inclusion of samples from two patients receiving broad-spectrum antibiotics mid-way through the study, may be an unavoidable confounder. Nevertheless, gut microbial analysis remained consistent across both exercising and control groups.

## Conclusions

The simple but important ‘take-home’ message for patients and their doctors is that short-term combined aerobic and resistance training will achieve favourable objective changes in body composition in patients with IBD and that this is not only safe, but also represents an inexpensive strategy for the prevention and treatment of IBD-related sarcopenia and obesity-related metabolic disorders. Policy- and guideline-makers should consider the addition of regular aerobic and resistance training to current treatment algorithms for patients with IBD. Patients should be reassured that physical activity does not aggravate IBD activity, particularly in exercise that is gradual and controlled. Moreover, this study addresses some of the unknowns relating to exercise in patients with IBD that have been highlighted previously by others [7, 11]. However, caution is required before advising patients with active disease; this requires more study and was not addressed in the current investigation.

## Additional files

Additional file 1: **Table S1.** Pre- and post-intervention values for resting inflammatory biomarkers in the exercise and control groups. (DOCX 16 kb)

Additional file 2: **Figure S1.** β-diversity of taxonomic profiles. A & B: Non-metric multidimensional scaling (NMDS) of relative abundance profiles for Bacteria species. C & D: Principal-coordinate analysis (PCoA) of Archaea relative abundance. (DOCX 626 kb)

Additional file 3: **Figure S2.** α-diversity of metabolic pathways. A to D: Shannon α-diversity H-index of general and species specific metabolic pathways at week 0 (pre) and week 8 (post). (DOCX 193 kb)

Additional file 4: **Figure S3.** β-diversity of metabolic pathways. (DOCX 581 kb)

## Abbreviations

1RM: One repetition maximum
BMI: Body Mass Index
DEXA: Dual Energy X-ray Absorptiometry
IBD: Inflammatory Bowel Disease
IQR: Interquartile Range
VO_2max_: Maximal capacity for oxygen consumption during aerobic exercise
