# Supplementary material for: Moderate-intensity aerobic and resistance exercise is safe and favorably influences body composition in patients with quiescent Inflammatory Bowel Disease: a randomized controlled cross-over trial
Source: BMC Gastroenterol. 2019 Feb 12;19:29. doi: 10.1186/s12876-019-0952-x (PMC6373036; doi:10.1186/s12876-019-0952-x)
Supplement: Supplementary file 1 — Table S1. Pre- and post-intervention values for resting inflammatory biomarkers in the exercise and control groups. (DOCX 16 kb) [file 12876_2019_952_MOESM1_ESM.docx]

**Additional file 1: Table S1.** Pre- and post-intervention values for resting inflammatory biomarkers in the exercise and control groups. There were no statistically significant differences detected for the change (Δ) in resting-state serum pro-inflammatory cytokines and C-reactive protein (CRP) between the exercise and control groups (Mann-Whitney U) after the study period. Median values (Interquartile ranges) are stated.

|  | **Pre-intervention** | **Post-intervention** | **Change (Δ)** | **p-value** | |
| --- | --- | --- | --- | --- | --- |
| **CRP (mg/L)**  *Exercise group*  *Control group* | 1 (1, 3.5)  1 (1, 2) | 1 (1, 2)  1 (1, 3) | 0 (0, 0)  0 (-0.75, 0.75) | 0.2 |  |
| **Interleukin-10 (pg/mL)**  *Exercise group*  *Control group* | 0.35 (0.31, 0.56)  0.45 (0.32, 0.86) | 0.39 (0.27, 0.88)  0.37 (0.33, 0.71) | 0.03 (-0.12, 0.18)  -0.12 (-0.27, 0.03) | 0.21 |  |
| **Interleukin-6**  **(pg/mL)**  *Exercise group*  *Control group* | 0.66 (0.59, 0.84)  0.57 (0.46, 1.71) | 0.6 (0.45, 1)  0.66 (0.55, 0.82) | 0.04 (-0.12, 0.2)  0.12 (0.06, 0.18) | 0.49 |  |
| **Interleukin-8**  **(pg/mL)**  *Exercise group*  *Control group* | 10.1 (7.56, 14.8)  12.25 (10.96, 19.46) | 10.64 (9.46, 16.26)  12.56 (10.06, 22.48) | 1.4 (-0.37, 3.17)  -4.17 (-8.2, -0.14) | 0.35 |  |
| **TNF-α**  **(pg/mL)**  *Exercise group*  *Control group* | 2.27 (1.87, 2.94)  2.32 (1.96, 2.8) | 2.4 (2.06, 3.2)  2.27 (1.9, 2.65) | 0.28 (0.05, 0.52)  -0.14 (-0.31, 0.03) | 0.18 |  |
