# Supplementary material for: Moderate-intensity aerobic and resistance exercise is safe and favorably influences body composition in patients with quiescent Inflammatory Bowel Disease: a randomized controlled cross-over trial
Source: BMC Gastroenterol. 2019 Feb 12;19:29. doi: 10.1186/s12876-019-0952-x (PMC6373036; doi:10.1186/s12876-019-0952-x)

**Additional file 2: Figure S1.** β-diversity of taxonomic profiles. (A & B) Non-metric multidimensional scaling (NMDS) of relative abundance profiles for Bacteria species demonstrates minimal shifts in diversity for Control (A) and Exercisers (B) following the 8 week treatment period. (C & D) Principal-coordinate analysis (PCoA) of Archaea relative abundance similarly shows subtle changes in the distribution of patients for both control (C) and exercise (D) groups. Statistical assessment of dissimilarity matrices was performed with the Adonis2 permutational multivariate analysis of variance (PERMANOVA) test. Density plots along the axes of all panels display the concentrations of data points, and were generated from kernel density estimates and scaled to a maximum value of 1.


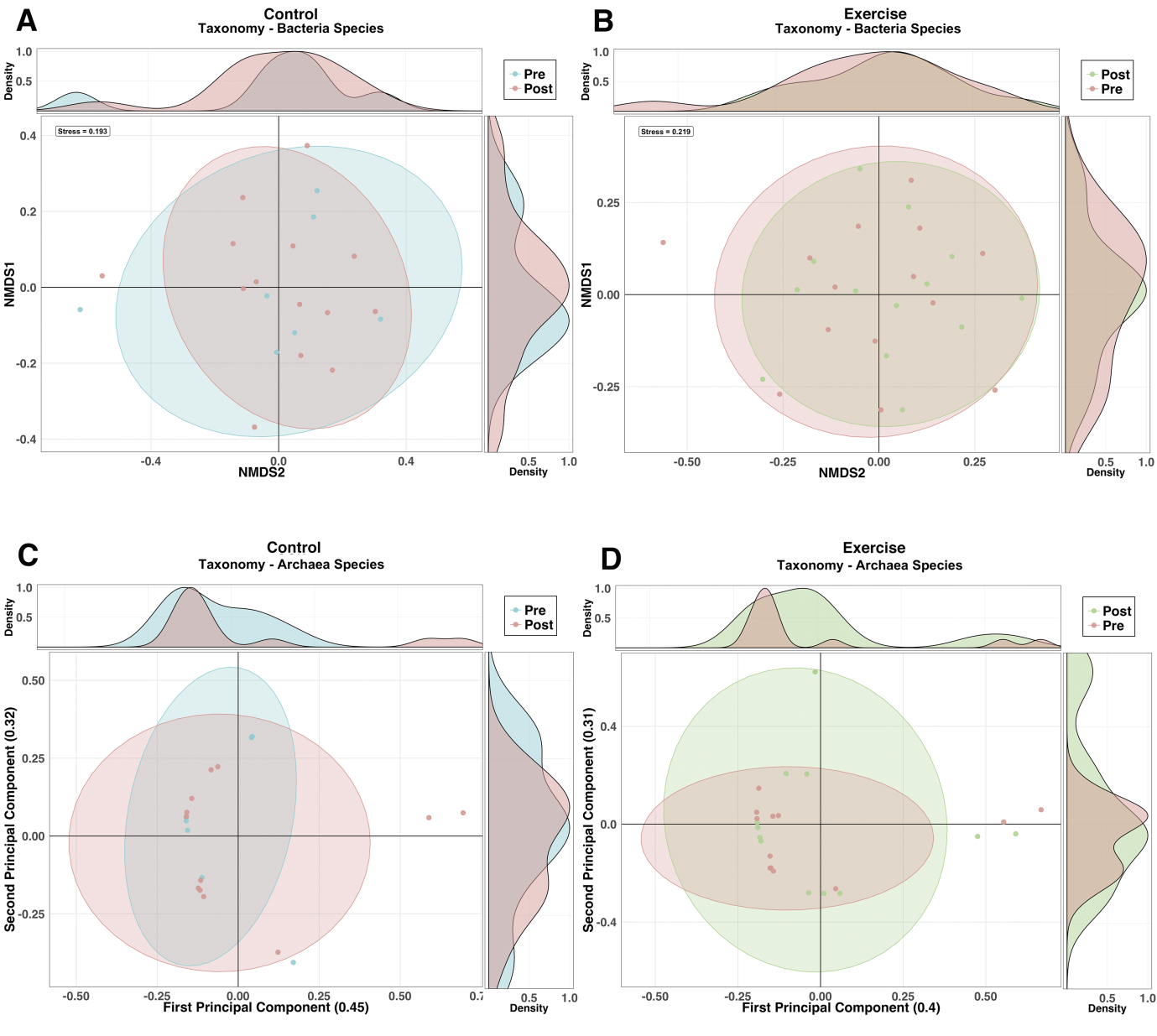

Supplement: Supplementary file 2 — Figure S1. β-diversity of taxonomic profiles. A & B: Non-metric multidimensional scaling (NMDS) of relative abundance profiles for Bacteria species. C & D: Principal-coordinate analysis (PCoA) of Archaea relative abundance. (DOCX 626 kb) [file 12876_2019_952_MOESM2_ESM.docx]
