# Supplementary material for: Moderate-intensity aerobic and resistance exercise is safe and favorably influences body composition in patients with quiescent Inflammatory Bowel Disease: a randomized controlled cross-over trial
Source: BMC Gastroenterol. 2019 Feb 12;19:29. doi: 10.1186/s12876-019-0952-x (PMC6373036; doi:10.1186/s12876-019-0952-x)

**Additional file 3: Figure S2**. α-diversity of metabolic pathways. (**A-D**) Shannon α-diversity H-index of general and species specific metabolic pathways at week 0 (pre) and week 8 (post). (**A** & **B**) No significant changes detected for general pathways of patients in either treatment. (**C** & **D**) A slight decrease of species specific pathways are shown in patients after 8 weeks of exercise. (**E**) Percent change (Δ) of α-diversity for general (**F**) and species specific pathways shows a slight increase in the exercise group for both, while a decrease is observed for the control group in species specific pathways. *P* values were calculated from the Wilcoxon signed-rank test.


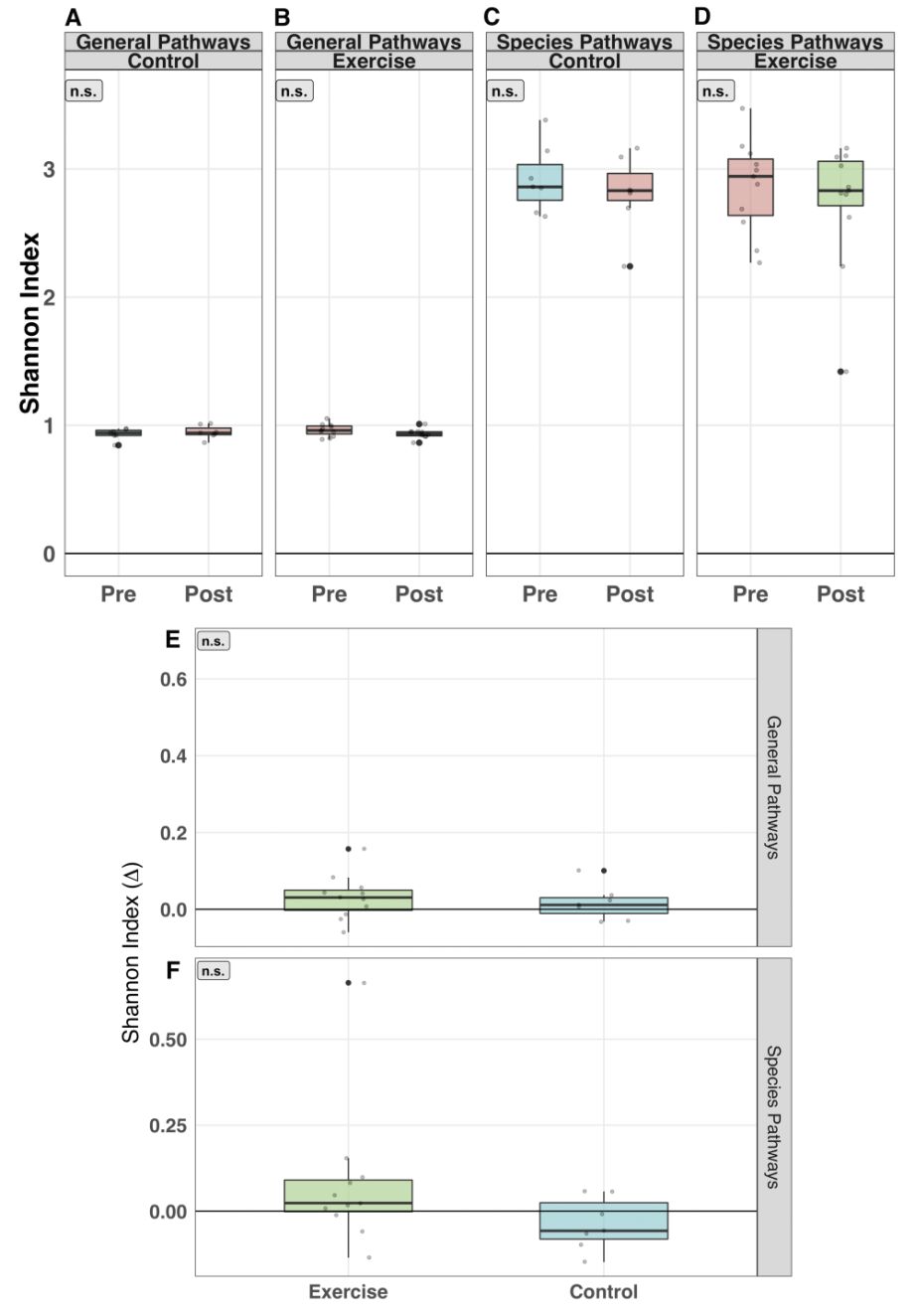

Supplement: Supplementary file 3 — Figure S2. α-diversity of metabolic pathways. A to D: Shannon α-diversity H-index of general and species specific metabolic pathways at week 0 (pre) and week 8 (post). (DOCX 193 kb) [file 12876_2019_952_MOESM3_ESM.docx]
