# Supplementary material for: Moderate-intensity aerobic and resistance exercise is safe and favorably influences body composition in patients with quiescent Inflammatory Bowel Disease: a randomized controlled cross-over trial
Source: BMC Gastroenterol. 2019 Feb 12;19:29. doi: 10.1186/s12876-019-0952-x (PMC6373036; doi:10.1186/s12876-019-0952-x)

**Additional file 4: Figure S3.** β-diversity of metabolic pathways. Demonstrating the distribution of patients in relation to the metabolic pathway models with non-metric multidimensional scaling (NMDS), no significant separations were detected. (**A** & **B**) Species specific pathways are minimally altered in diversity for Control (**A**) and Exercise (**B**) groups following the 8 week treatment period. (**C** & **D**) Unique pathways similarly had subtle changes in distribution for patients of both Control (**C**) and Exercise (**D**) groups. Statistical assessment of dissimilarity matrices was performed with the Adonis2 permutational multivariate analysis of variance (PERMANOVA) test. Density plots along the axes of all panels display the concentrations of data points, and were generated from kernel density estimates and scaled to a maximum value of 1.


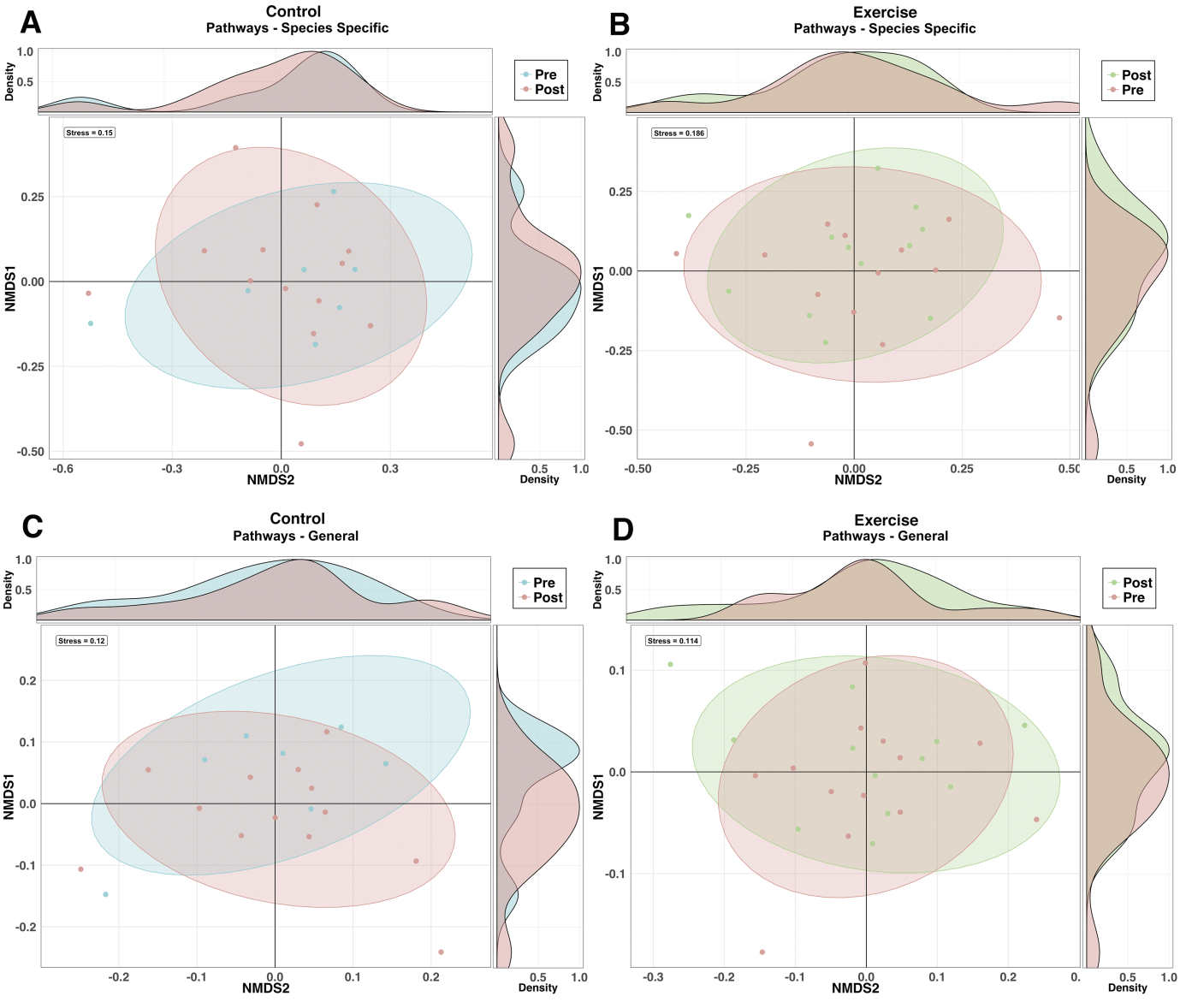

Supplement: Supplementary file 4 — Figure S3. β-diversity of metabolic pathways. (DOCX 581 kb) [file 12876_2019_952_MOESM4_ESM.docx]
